# Intra‐rater and inter‐rater reliability of robotic arthrometer DYNEELAX®

**DOI:** 10.1002/jeo2.70026

**Published:** 2024-09-30

**Authors:** Katja Mihalinec, Carmen B. Martinez‐Cepa, Juan C. Zuil‐Escobar, Nataša Kejžar, Renata Vauhnik

**Affiliations:** ^1^ Department of Physiotherapy Biomechanical Laboratory, Faculty of Health Sciences, University of Ljubljana Ljubljana Slovenia; ^2^ Departamento de Fisioterapia Facultad de Medicina, Urbanización Montepríncipe, Universidad San Pablo‐CEU, CEU Universities Boadilla del Monte Spain; ^3^ Faculty of Medicine, Institute for Biostatistics and Medical Informatics University of Ljubljana Ljubljana Slovenia; ^4^ Arthron, Institute for Joint and Sports Injuries, Ukmarjeva 2 Ljubljana Slovenia

**Keywords:** anterior laxity, arthrometry, rotational laxity, reliability

## Abstract

**Purpose:**

The purpose of our study was to test the intra‐rater and inter‐rater reliability of the DYNEELAX® arthrometer in healthy subjects. Since rotational laxity will be measured for the first time in humans, indications for normative values will also be presented.

**Methods:**

Knee anterior laxity and rotational laxity using a DYNEELAX® arthrometer were assessed in 73 subjects (39 females and 34 males). An intraclass correlation coefficient was calculated to evaluate the intra‐rater and inter‐rater reliability of the DYNEELAX®.

**Results:**

An intraclass correlation coefficient for intra‐rater reliability ranges from 0.631 (95% confidence interval; [CI]: 0.47–0.75) to 0.699 (95% CI: 0.56–0.80) and from 0.916 (95% CI: 0.87–0.95) to 0.94 (95% CI: 0.91–0.96) for anterior knee laxity and rotational knee laxity, respectively. An intraclass correlation coefficient for inter‐rater reliability ranges from 0.578 (95% CI: 0.40–0.71) to 0.646 (95% CI: 0.44–0.78) and from 0.822 (95% CI: 0.57–0.91) to 0.933 (95% CI: 0.89–0.96) for anterior knee laxity and rotational knee laxity, respectively.

**Conclusions:**

The DYNEELAX® arthrometer has good to excellent intra‐rater and inter‐rater reliability for rotational knee laxity and moderate intra‐rater reliability for anterior knee laxity in healthy subjects. Future studies should investigate the clinical significance of anterior and rotational laxity measured with the DYNEELAX® arthrometer in patients with knee pathology, as both laxities are critical for assessing the integrity of the intra‐articular structures of the knee in clinical practice.

**Level of evidence:**

Level IV.

**Abbreviations**
ACL: anterior cruciate ligament
ER: external rotation
ICC: intraclass correlation coefficient
IQR: interquartile range
IR: internal rotation
MDC: minimal detectable change
SD: standard deviation
SEM: standard error of measurement

## INTRODUCTION

Increased knee laxity is an important risk factor to consider when dealing with knee ligament injuries or traumatic knee injuries [16, 30], especially when linked to an anterior cruciate ligament (ACL) that can result in osteoarthritic changes in the knee, starting a few years after the injury [13]. This leads to the reduced quality of life of the injured person in the long term due to the inability of participating in activities done before the injury [8]. However, not only increased anterior knee laxity but also rotational laxity when present can lead to instability and may be one of the biggest factors leading to a delayed return to sports activity in both amateur and professional athletes [9, 24]. Rotational laxity itself can result in functional deficits, as a result of a single or multi‐ligament injury [25]. ACL is known to be resistant to anterior knee laxity, but adding rotational components can better describe its integrity. It was found that the ACL resists rotational motion, more significantly internal rotation (IR) of the tibia against the femur [3, 4]. This states that anterior laxity testing alone without also testing the rotational motion may not be sufficient to address the functional deficits, which are clinically still difficult to test in an objective way because no such arthrometer has been available [24]. Another important ligament for rotational stability is the anterolateral ligament (ALL) which is clinically tested with a pivot shift test [10]. There are some devices for isolated passive rotational motion reported in the literature like the Rotameter [14], Rottometer [2], Robotic Knee Testing Tool [5] and Vermont knee laxity device (also for testing in varus–valgus direction) [22]. However, arthrometers measuring rotational movement in isolation were discussed as not being significant enough for ACL integrity evaluation because there is no anterior laxity component included in the test [1, 14].

An arthrometer (DYNEELAX®) that can measure anterior laxity and rotational laxity has been developed. To our knowledge, there was no device previously described in the literature to measure laxity first in the sagittal and then in the axial planes within one device, without moving the leg being tested between measurements. The DYNEELAX®'s prototype was first described by Cojean et al. in 2023 [7] with a study made on the prototyped leg. It was found that DYNEELAX®'s repeatability is excellent when respecting parameters such as positioning patellar and ankle support and most importantly patellar tightening. Since no study with the same arthrometer has been done on humans to assess the reliability, the aim of our study was to test the intra‐rater and inter‐rater reliability of the DYNEELAX® arthrometer. Reliability of the device is crucial for the use of devices in a clinical environment. Since rotational laxity will be measured for the first time in humans, indications for normative values will also be presented. Our hypothesis is that the DYNEELAX® arthrometer has excellent intra‐rater and inter‐rater reliability and represents a reliable evaluation tool.

## METHODS

### Participants

Participants were healthy adults, aged between 18 and 60 years. Subjects who had a history of a traumatic knee injury or surgery of the knee were excluded. Before participating in the research, the purpose of the research was explained and a consent form for participation in the research was signed. The research was approved by the Committee for Medical Ethics of the Republic of Slovenia (Number of approval: 0120‐513/2022/3).

### Testing protocol

The study followed a test–retest reliability design for intra‐rater reliability study and the second examiner for the inter‐rater reliability study made one additional test. Testing was performed three times, with females having to be retested 24 h after the first test and males within 7 days in intra‐rater reliability study. Females were retested in 24 h in an attempt to limit the effects of changes in female hormone levels on knee laxity [21]. Randomisation of the first tested leg and the tester was performed with the R programme [17] in advance for all participants. The first tester was a physiotherapist with 1 year of clinical experience, inexperienced in using the DYNEELAX® arthrometer, and the second tester participating only in inter‐rater reliability study was a physiotherapist also inexperienced in the use of the device. Both underwent training in handling the arthrometer before the study with a pilot study. All tests were performed using the same protocol for DYNEELAX® testing, proposed and standardised by the manufacturer (Genourob®).

**Figure 1:**
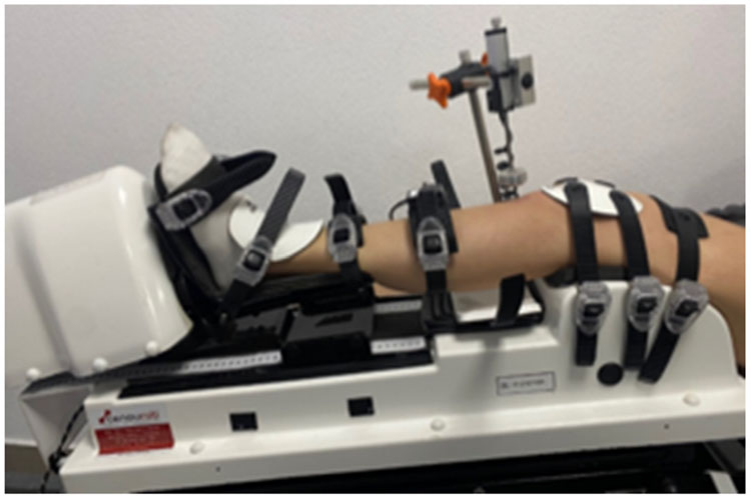
Left leg on the DYNEELAX®.

Participants were positioned supine with arms next to the body on the therapeutic table designed for DYNEELAX® testing, with the trunk being inclined. The tested leg was positioned on a rigid adjustable leg support with the knee 20° flexed for all the tests performed (Figure 1). The DYNEELAX® can gradually produce forces from 0 to 300 N by pushing the posterior aspect of the calf into the anterior direction. The first sensor for measuring anterior laxity in millimetres was placed on the tibia tuberosity, perpendicularly on the tibia. The DYNEELAX® is also performing tibial rotations with torques from 1 to 10 Nm. The second sensor, measuring the rotational movement of the tibia in degrees (rotational laxity), was placed on the anterior aspect of the tibia (in the middle third of the tibia) with a strap around the calf. The neutral position of the second sensor, which was placed directly on the skin, is secured by respecting the angle of the sensor that is seen in the software programme on the computer (in the zone from −1 to +1).

The following study testing protocol was used, for anterior knee laxity: one repetition with 134 N, one repetition with 150 N, and three repetitions with 200 N, to measure the displacement of the tibia with respect to the femur. For rotational laxity, the protocol with one repetition with 3 Nm and three repetitions with 5 Nm torques was used.

Leg fixation on the device started distally, by tightening ankle straps (first, the proximal strap, then the distal one, and lastly the middle one) and was followed by positioning of the two sensors. Just before the start of the measurement femur stabilisation, and patella stabilisation were made. Patellar stabilisation needed to be greater than 80 N (80–100 N, if possible). Patellar tightening was standardised and did not differ by more than 10 N, between the left and right leg, as well as between tests. All the data were recorded in software on the computer. Each measurement session lasted between 20 and 30 min.

Leg dominance was assessed using two tests at the beginning of the first session for each participant. First, the test to kick a ball was performed where the dominant leg is the leg that is used to kick a ball. The second test was a test where a push of a participant's upper body is made from behind which forces a person to make a step to prevent a fall and the leg chosen for the step is considered as the dominant leg [6, 28]. If the results of the two tests described varied, the first test (ball kicking) was repeated once again and used to determine the dominant leg.

### Statistical analysis

Statistical analysis was done with the R programme [17]. Descriptive statistics of means and standard deviations (SD) or medians and interquartile ranges (IQR) were reported for symmetrical and asymmetrical numerical variables, respectively, and frequencies (percentages) were reported for categorical variables. For all analyses, a 95% level of confidence was chosen. Intraclass correlation coefficients (ICC) were calculated for anterior laxity at 134 and 200 N and external rotation (ER) and internal rotation (IR) at 5 Nm. The ICC model (1,1) has been chosen for the intra‐rater reliability and ICC model (2,1) has been chosen for the inter‐rater reliability. Both models were selected based on our type of data [20, 32]. Reliability was categorised as ICC: <0.5 (poor reliability); 0.5–0.75 (moderate reliability); 0.75–0.9 (good reliability); and >0.90 (excellent reliability) [12]. In addition to the ICC, the standard errors of measurement (SEM) and the minimal detectable changes (MDC) were calculated. ICC values were complemented with Bland Altman plots, where the variance of measurements can also be observed. Pearson correlation coefficients were calculated to see whether there is a correlation between rotational laxity variables and their correlation to the anterior laxity variables.

## RESULTS

Seventy‐three participants (39 females, 34 males), aged between 18 and 49 years, with a median and IQR of 22 (8) years, participated in the study. The demographic data from the participants are presented in Table 1.

**Table 1:** Demographic data (*N* = 73).

	Female	Male	All
Number of participants, *n* (%)	39 (53%)	34 (47%)	73
Age (years), median (IQR)	21.0 (6.0)	23.5 (8.0)	22.0 (8.0)
Body height (cm), mean (SD)	164.0 (6.3)	180.3 (7.0)	171.6 (10.5)
Body mass (kg), mean (SD)	58.8 (7.6)	78.3 (10.0)	67.9 (13.1)
BMI (kgm^2^), mean (SD)	21.9 (2.5)	24.1 (2.5)	22.9 (2.7)
Leg dominance – Right, *n*	33	29	62
Leg dominance – Left, *n*	6	5	11
Oral contraception, *n* (%)	12 (31%)	/	/
Regular menstrual cycle, *n* (%)	31 (80%)	/	/
Premenstrual symptoms, *n* (%)	20 (51%)	/	/

Abbreviations: BMI, body mass index; IQR, interquartile range; SD, standard deviation.

The average values of anterior knee laxity and rotational laxity, for Test 1 and Test 2 are presented in Table 2.

**Table 2:** Mean (SD) laxity values for all the tests.

Knee laxity	Female Mean (SD)	Male Mean (SD)	All Mean (SD)
Anterior laxity test 1, 134 N force on left (mm)	3.66 (0.63)	3.70 (0.94)	3.68 (0.79)
Anterior laxity test 2, 134 N force on left (mm)	3.66 (0.55)^a^	3.86 (1.09)	3.76 (0.85)^a^
Anterior laxity test 1, 134 N force on right (mm)	3.62 (0.50)	3.43 (0.68)	3.53 (0.59)
Anterior laxity test 2, 134 N force on right (mm)	3.63 (0.56)^a^	3.38 (0.48)	3.51 (0.54)^a^
Anterior laxity test 1, 200 N force on left (mm)	5.27 (0.73)^a^	5.24 (1.07)	5.26 (0.90)^a^
Anterior laxity test 2, 200 N force on left (mm)	5.32 (0.65)^a^	5.25 (0.99)	5.29 (0.82)^a^
Anterior laxity test 1, 200 N force on right (mm)	5.26 (0.71)^a^	5.75 (0.83)	5.11 (0.78)^a^
Anterior laxity test 2, 200 N force on right (mm)	5.28 (0.80)^b^	4.95 (0.83)	5.10 (0.73)^b^
Rotational laxity test 1 for ER, 5 N force on left (°)	15.55 (3.8)	8.10 (2.44)	12.08 (4.94)
Rotational laxity test 2 for ER, 5 N force on left (°)	15.32 (4.14)^a^	7.01 (2.13)	11.77 (5.03)^a^
Rotational laxity test 1 for ER, 5 N force on right (°)	15.88 (4.45)	8.25 (2.55)	12.33 (5.30)
Rotational laxity test 2 for ER, 5 N force on right (°)	16.05 (4.0)^a^	8.28 (2.24)	12.39 (5.10)^a^
Rotational laxity test 1 for IR, 5 N force on left (°)	14.11 (3.79)	8.13 (2.59)	11.33 (4.43)
Rotational laxity test 2 for IR, 5 N force on left (°)	13.99 (4.07)^a^	7.96 (2.63)	11.14 (4.59)^a^
Rotational laxity test 1 for IR, 5 N force on right (°)	13.19 (3.98)	7.39 (2.18)	10.49 (4.36)
Rotational laxity test 2 for IR, 5 N force on right (°)	13.08 (3.26)^a^	7.50 (2.15)	10.44 (3.94)^a^

Abbreviations: ER, external rotation; IR, internal rotation.

^a^ No results for one participant.

^b^ No result for two participants.

Indications for normative values (based on the sample of 72 subjects) for rotational laxity were taken from the second part of Table 2, separately for males and females. Normative data for rotational laxity in the direction of ER can be considered between 15.3° and 16.1° (with an SD of 4.5°) for females and from between 7° and 8.5° (with an SD of 2.6°) for males. The values for rotational laxity in the direction of IR can be considered from 13° to 14.1° (with an SD of 4.1°) for females and for males from 7.4° to 8.2° (with an SD of 2.6°).

In Table 3, ICC, SEM and MDC values for intra‐rater reliability are reported. In Table 4, ICC, SEM and MDC values for the inter‐rater reliability study are reported.

**Table 3:** Intra‐rater reliability.

Knee laxity test	ICC	95% CI	SEM	MDC 95%
Anterior laxity test, 134 N force on left	0.631	0.471–0.751	0.498	1.381
Anterior laxity test, 134 N force on right	0.699	0.561–0.800	0.311	0.863
Anterior laxity test, 200 N force on left	0.71	0.575–0.807	0.466	1.293
Anterior laxity test, 200 N force on right	0.681	0.536–0.787	0.429	1.189
Rotational laxity test for ER, 5 N force on left	0.919	0.874–0.958	1.423	3.943
Rotational laxity test for ER, 5 N force on right	0.94	0.907–0.962	1.375	3.535
Rotational laxity test for IR, 5 N force on left	0.929	0.890–0.955	1.21	3.354
Rotational laxity test for IR, 5 N force on right	0.916	0.869–0.946	1.218	3.375

Abbreviations: CI, confidence interval; ER, external rotation; ICC, intraclass correlation coefficient; IR, internal rotation; MDC, minimal detectable change; SEM, standard error of measurement.

**Table 4:** Inter‐rater reliability.

Knee laxity test	ICC	95% CI	SEM	MDC 95%
Anterior laxity test, 134 N force on left	0.646	0.440–0.778	0.487	1.349
Anterior laxity test, 134 N force on right	0.578	0.403–0.712	0.405	1.122
Anterior laxity test, 200 N force on left	0.602	0.377–0.748	0.597	1.655
Anterior laxity test, 200 N force on right	0.615	0.450–0.739	0.498	1.380
Rotational laxity test for ER, 5 N force on left	0.854	0.778–0.906	2.007	5.563
Rotational laxity test for ER, 5 N force on right	0.933	0.891–0.959	1.421	3.939
Rotational laxity test for IR, 5 N force on left	0.822	0.569–0.913	1.741	4.825
Rotational laxity test for IR, 5 N force on right	0.931	0.892–0.956	1.184	3.283

Abbreviations: CI, confidence interval; ER, external rotation; ICC, intraclass correlation coefficient; IR, internal rotation; MDC, minimal detectable change; SEM, standard error of measurement.

**Figure 2:**
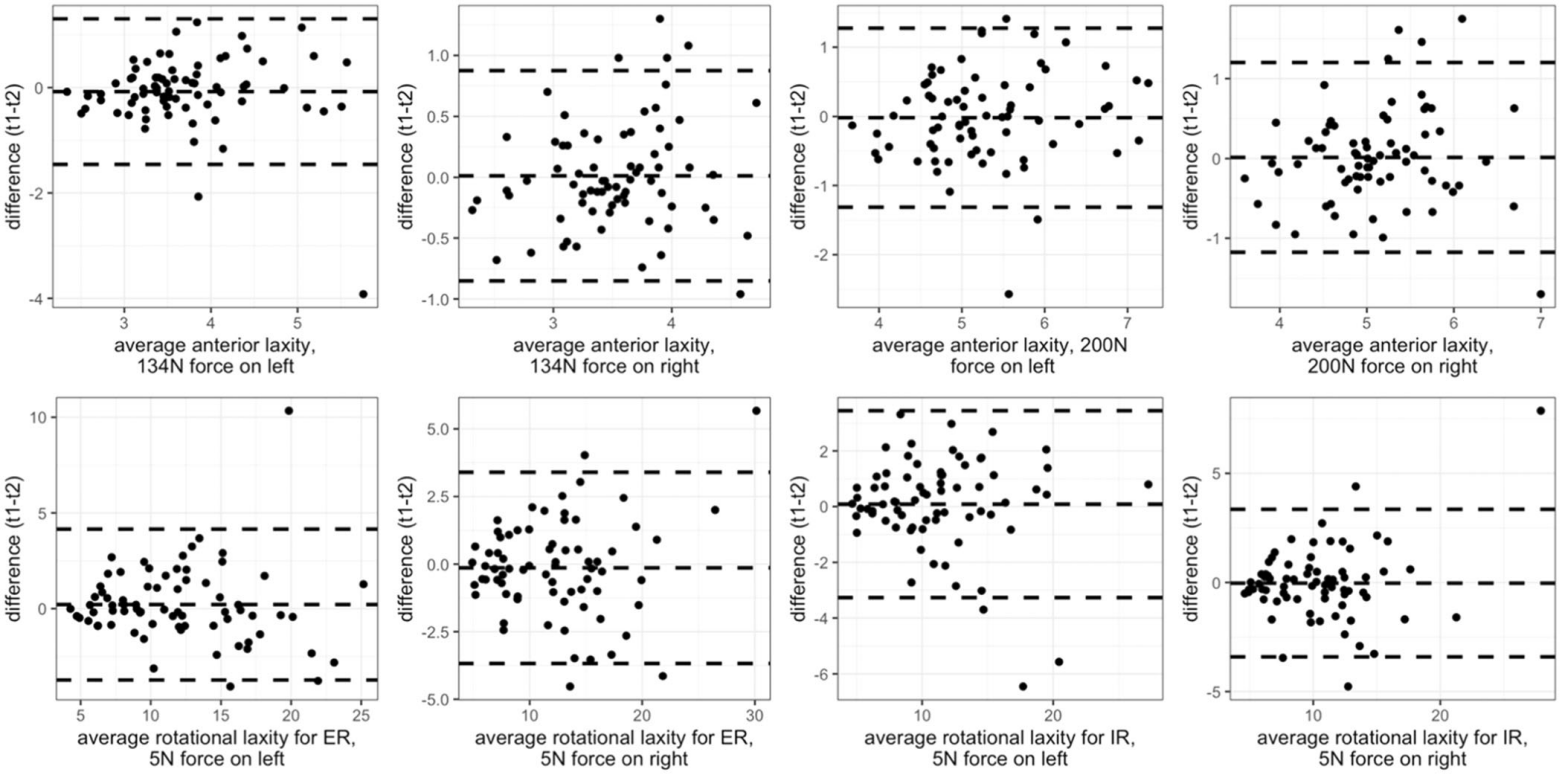
Bland–Altman plots for the difference between Test 1 and Test 2 (t1 − t2).

With Bland–Altman plots no learning effects were observed or trends in the variance of measurements. Some outliers were found, mostly on the left leg, but for different subjects (therefore none was systematic) (Figure 2)*.*

**Figure 3:**
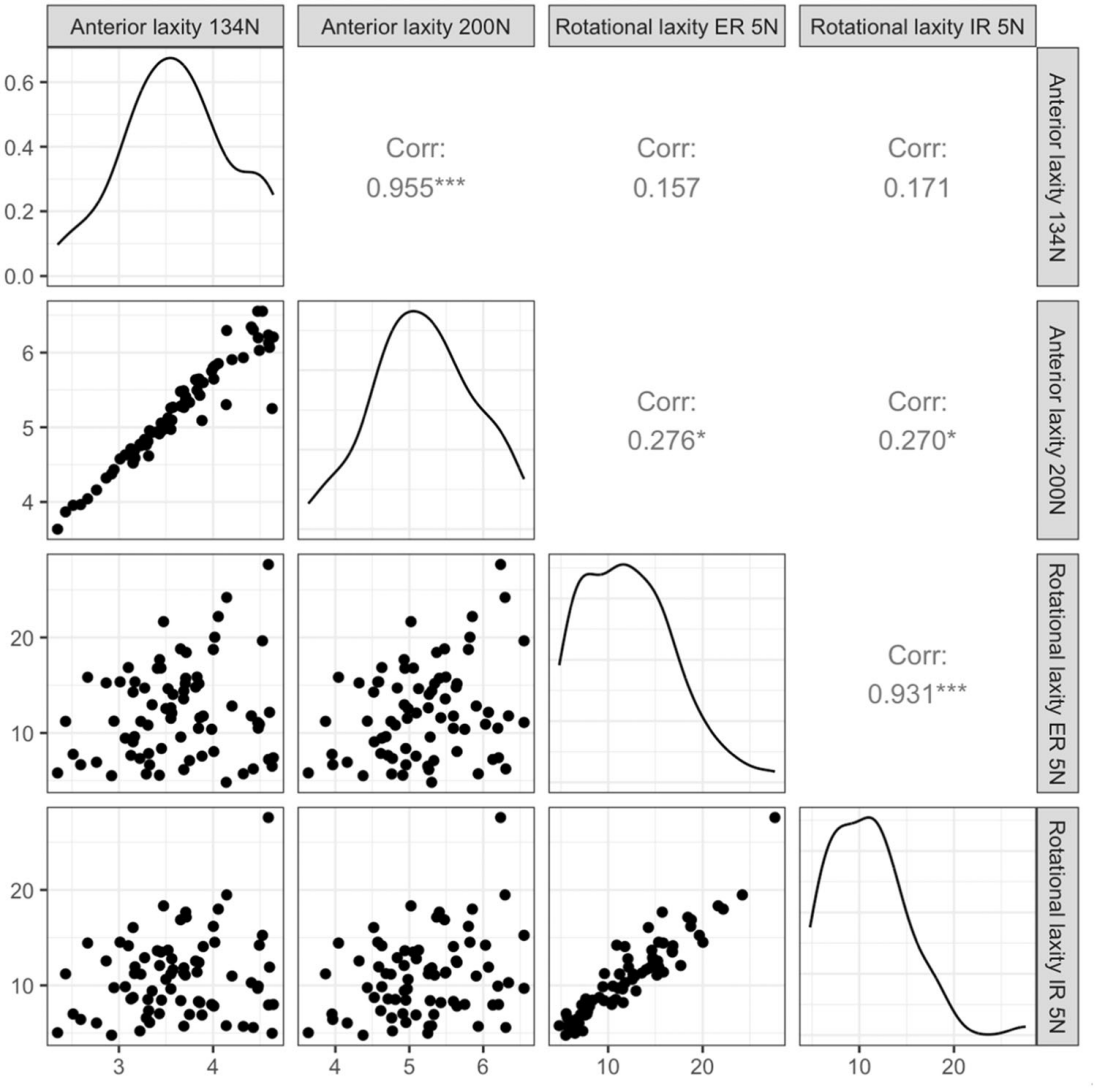
Upper triangle: Pearson correlation coefficient between average measurements (* denotes *p* < 0.05, *** denotes *p* < 0.001), lower triangle: scatterplots, diagonal: empirical distribution of a variable.

A correlation analysis between rotational laxity and anterior laxity was made (Figure 3). Pearson correlation coefficients for anterior laxity on 134 N with ER: 0.157, for anterior laxity on 200 N with ER: 0.276, for anterior laxity on 134 N with IR: 0.171, with anterior laxity on 200 N with IR: 0.270. There is a probable correlation between rotational laxity (IR and ER) with anterior laxity test on 200 N (*p* < 0.05); however, all the sample correlations are low (i.e., <0.3). Additionally, a high positive Pearson correlation coefficient of 0.931 between rotational laxity in IR and ER was observed.

## DISCUSSION

The DYNEELAX® arthrometer has moderate intra‐rater and inter‐rater reliability for anterior knee laxity and good to excellent intra‐rater and inter‐rater reliability for rotational laxity in healthy subjects. As this is the first study to evaluate the reliability of the DYNEELAX®, a comparison of the results with other reliability studies of the same device used on humans cannot be made. However, as this is currently the only device that can measure both anterior tibial translation and tibial rotation, it has important clinical implications as the assessment of both can provide a more precise and accurate picture of the integrity of the intra‐articular structures of the knee joint. The DYNEELAX® arthrometer and its predecessor, the GNRB®, appear to have similar reliability for the assessment of anterior knee laxity. Smith et al. [26] found that both intra‐rater and inter‐rater reliability of anterior knee laxity evaluation with GNRB® was moderate to good (ICC: 0.72–0.83 and ICC: 0.76–0.84 for intra‐rater and inter‐rater reliability, respectively). The ICC values in their study were overall higher in studying intra‐rater reliability than in the data set (ICC: 0.631–0.71) and in previous work by Vauhnik et al. [31]. While the inter‐rater results (ICC: 0.578−0.646) were similar in our study and in the study of Smith et al. [26], Vauhnik et al. [29] found inferior results (ICC: 0.220–0.424). In the most recent study examining intra‐rater reliability of the GNRB in 97 participants involved in different sport activities, Magdič et al. [15] reported good intra‐rater reliability (ICC: 0.756–0.848). In the mentioned study, the reliability for anterior laxity test might be better than using DYNEELAX® because they have performed an electromyographic evaluation of hamstring muscle activation during the test. This could be one of the factors contributing to the differences in the reliability results, as the hamstrings are known to work in synchrony with anterior cruciate ligament and their contraction could lead to less anterior tibial translation [27]. In our sample, some participants were noted to have respect for testing, especially in their first testing session, which could lead them to unconsciously contract hamstring to feel in control of the test. This is based on our observation that stabilising force values displayed in the arthrometer software were affected during the anterior laxity test and were therefore difficult to adjust. However, there does not appear to be a learning effect from the first to second measurement in our study. Nevertheless, we recommend performing an electromyographic evaluation of the hamstrings when working with the DYNEELAX® to ensure that the participants are relaxed during test. Regarding patellar stabilisation force we also observed that the force proposed by the manufacturer (80–100 N) was challenging to achieve in smaller participants (especially those with lower weight), as it could be painful for the participant or compress the muscles and this trigger their activation. This is in agreement with Magdič et al. [15] who discussed the problem when testing overweight participants not only in terms of stabilisation but also because of the thicker tissue under both sensors, which seems to affect the results. To our knowledge, there is no inter‐rater reliability study for the devices measuring rotational laxity.

In addition to the ICC values, the standard error of measurement (SEM) and the minimal detectable change (MDC) were calculated. The observed MDC is mostly smaller than the clinically important change for the measurement of anterior laxity of 1.5 mm, which would represent a partial ACL tear in the GNRB test [18]. In both tests for anterior laxity (134 and 200 N), a slightly larger MDC is seen on the left leg. As there are not many outliers this could be purely coincidental to be linked to the examiner's dominant hand, which was right for both examiners and could have influenced the performance of the test on the left leg. Similarly, hand dominance influence was already commented on in the study published in 2013 by Vauhnik et al. [31] who found a difference in reliability between the legs tested (better for right leg). It is not clear why hand dominance could have an influence on testing with robotic devices such as DYNEELAX® or GNRB®, as has been found for other manual arthrometers [19]. Furthermore, since different tibial rotation during leg positioning can affect the amount of laxity, this could also explain the outliers found in the present study. On the other hand, the inter‐rater study shows slightly higher MDC values than the intra‐rater study, especially for the anterior laxity test with 200 N, again on the left leg, which is higher (1.66) than the clinically important change of 1.5 mm. For rotational laxity, MDC values were smaller (MDC: 3.35–3.38 for IR and MDC: 3.54–3.94 for ER) than true side to side difference (4°−7°) and less than or equal to the clinically important difference (3°–4°) calculated based on measurements with Vermont device [22]. The MDC values of rotational laxity are higher in the intra‐rater reliability than in the inter‐rater reliability. From small 95% MDC values, we can conclude that the differences between tests done by the same examiner are probably not large enough to be clinically important, for anterior knee laxity nor for rotational knee laxity testing. However, this cannot be said for tests performed by different examiners, as the MDC results are slightly higher for the left leg, indicating a potential difference between the sides.

Since the ACL not only prevents anterior tibial translation but also tibial rotation [4, 11], mostly not in isolation but in synchronised restriction with other collateral structures of the knee [4, 33] we investigated the possible correlations between anterior tibial translation and tibial rotation. Based on Pearson correlation coefficients, weak positive relationships between anterior tibial translation and tibial rotation are evident. However, correlations appear to be too small to have a likely clinical importance. On the other hand, we found a strong correlation between IR and ER rotational laxity with a Pearson correlation coefficient of 0.931. Shultz et al. [23] found a correlation between anterior and rotational knee laxity (0.313–0.610) that is slightly stronger than in the present study (and an even stronger correlation of anterior knee laxity to valgus‐varus motion). The main limitation of our study is that the subjects included for testing the intra‐rater and inter‐rater reliability of the DYNEELAX® arthrometer were healthy individuals without knee pathology and therefore we cannot discuss the results in terms of their clinical significance.

## CONCLUSIONS

This study investigated the intra‐rater and inter‐rater reliability of the DYNEELAX® arthrometer in healthy subjects. This device has good to excellent intra‐rater and inter‐rater reliability for rotational knee laxity and moderate intra‐rater reliability for anterior knee laxity in healthy subjects. As the assessment of the integrity of the intra‐articular structures of the knee is of crucial importance in clinical practice, future studies should investigate the clinical significance of anterior and rotational laxity measured with this device in patients with knee pathology.

## AUTHOR CONTRIBUTIONS

All authors have made substantial contributions to all of the following: (1) the conception and design of the study, or acquisition of data, or analysis and interpretation of data, (2) drafting the article or revising it critically for important intellectual content, (3) final approval of the version to be submitted. All the authors have read and concurred with the content in the final manuscript.

## CONFLICT OF INTEREST STATEMENT

The authors declare no conflict of interest.

## ETHICS STATEMENT

The research was approved by the Committee for Medical Ethics of the Republic of Slovenia (Number of approval: 0120‐513/2022/3).

## Data Availability

The raw measurements are available in Supplemental File.
